# Acceptability of self-collection sampling for HPV-DNA testing in low-resource settings: a mixed methods approach

**DOI:** 10.1186/1471-2458-14-596

**Published:** 2014-06-12

**Authors:** Pooja Bansil, Scott Wittet, Jeanette L Lim, Jennifer L Winkler, Proma Paul, Jose Jeronimo

**Affiliations:** 1 PATH, Seattle, Washington, USA

**Keywords:** Self-sampling, Experiences, Cervical cancer screening, Human papillomavirus (HPV), Low income resource settings

## Abstract

**Background:**

Vaginal self-sampling with HPV-DNA tests is a promising primary screening method for cervical cancer. However, women’s experiences, concerns and the acceptability of such tests in low-resource settings remain unknown.

**Methods:**

In India, Nicaragua, and Uganda, a mixed-method design was used to collect data from surveys (N = 3,863), qualitative interviews (N = 72; 20 providers and 52 women) and focus groups (N = 30 women) on women’s and providers’ experiences with self-sampling, women’s opinions of sampling at home, and their future needs.

**Results:**

Among surveyed women, 90% provided a self- collected sample. Of these, 75% reported it was easy, although 52% were initially concerned about hurting themselves and 24% were worried about not getting a good sample. Most surveyed women preferred self-sampling (78%). However it was not clear if they responded to the privacy of self-sampling or the convenience of avoiding a pelvic examination, or both. In follow-up interviews, most women reported that they didn’t mind self-sampling, but many preferred to have a provider collect the vaginal sample. Most women also preferred clinic-based screening (as opposed to home-based self-sampling), because the sample could be collected by a provider, women could receive treatment if needed, and the clinic was sanitary and provided privacy. Self-sampling acceptability was higher when providers prepared women through education, allowed women to examine the collection brush, and were present during the self-collection process. Among survey respondents, aids that would facilitate self-sampling in the future were: staff help (53%), additional images in the illustrated instructions (31%), and a chance to practice beforehand with a doll/model (26%).

**Conclusion:**

Self-and vaginal-sampling are widely acceptable among women in low-resource settings. Providers have a unique opportunity to educate and prepare women for self-sampling and be flexible in accommodating women’s preference for self-sampling.

## Background

Cervical cancer is largely preventable. However, it is the third most common cancer among women worldwide, and poses a public health problem in developing countries where 85% of the global deaths due to cervical cancer occur [1]. Cervical cancer-related deaths have decreased significantly in developed countries due to widespread screening based on Pap smear testing. However, similar initiatives in developing countries have not had the same success due to the complexity of the elements required. The success of Pap smear testing depends on repeat testing and high-quality laboratories, which drive up costs [2]. Additionally, the lack of trained personnel who can adequately read cytology samples leads to a long waiting time (1 to 3 months) for receiving test results, causing high loss to follow-up [3].

As the sensitivity of speculum based Pap smears for detecting moderate to severe cervical intraepithelial neoplasia (CIN) or cancer has been shown to be sub-optimal (50%) even in very high quality labs [4,5] the use of HPV-DNA testing seems promising as a primary screening method in low-resource settings [6-9]. *Care*HPV™ is a new HPV-DNA test that is ideal for low income resource setting as it is cost-effective (lower cost per test) as compared to other HPV-DNA tests, simple to use by laboratory technical staff and can provide rapid results within 3 hours [9]. *Care*HPV™ can be performed on both provider- and self-collected samples, and findings show that the clinical performance for detecting cervical intraepithelial neoplasia grade 2 or more severe diagnosis (CIN2+) was comparable to other HPV-DNA screening tests such as Hybrid Capture 2 [3,9].

Although recent studies have shown that provider-collected cervical samples (collected during a pelvic exam) resulted in the highest HPV-DNA sensitivity, ranging between 84 and 100%, the sensitivity of self-collected vaginal HPV-DNA tests ranged between 66 and 88%; both had similar specificities [10]. Moreover, findings from recent systematic reviews show that self-sampling was highly acceptable and that a majority of women preferred vaginal self-sampling to provider-collected cervical sampling [11,12]. As compared to the conventional speculum based Pap smear procedure, women preferred self-sampling because it was private, comfortable, less painful, and less embarrassing [13,14]. Moreover, research indicates that the correlation between self-collected and provider-collected samples was good [10,15]. In light of these results and given that self-sampling does not require a pelvic exam with speculum, it has the potential to be a more acceptable screening option for women, especially in low-resource settings where there are logistical limitations to performing the exam, or in areas where there are cultural barriers to conducting a pelvic exam [3].

Several studies have evaluated women’s opinions of self-sampling [13,16-19]. However, to date, no studies have provided a broad and in-depth examination of women’s perspectives about self-sampling acceptability especially in low-resource settings, using both quantitative and qualitative research methods. Thus, the objectives of this study were to gain further insight into: 1) women’s experiences and concerns with cervical cancer screening ; 2) women’s experiences and concerns with self-sampling; 3) providers’ experiences of self-sampling; 4) women’s experiences with self-sampling at home; and 5) how to facilitate self-sampling in the future (e.g., guidance in training or messaging).

## Methods

This cross-sectional mixed-method study was implemented within the context of a cervical cancer screening demonstration project, The Screening Technologies to Advance Rapid Testing—Utility and Program Planning (START-UP) project in India, Nicaragua, and Uganda, the main objective of which was to generate evidence comparing various screening options implemented by public health systems in regionally representative developing-country settings [20]. This study was conducted in accordance with the Helsinki Declaration; study protocols for each site were similar and were approved by local and regional Institutional Review Boards in all project sites (Nicaragua: Country IRB, Research Division - Minister of Health; Uganda: Makerere University Ethics Committee; India: Institute for Cytology and Preventive Oncology ethics committee, MNJ Institute of Oncology and Regional Cancer Center, Institute Ethics Committee), and by PATH’s Research and Ethics Committee.

As part of the demonstration project, eligible women that gave written consent were invited to self-collect a vaginal *care*HPV™ (HPV-DNA) sample during their initial screening visit. If a woman chose not to self-sample, a provider offered to collect the vaginal sample for her. Subsequently, all enrolled women received a pelvic examination (with speculum) during which a provider collected a cervical *care*HPV™ sample and a cervical Pap smear sample, and then performed visual screening with acetic acid (VIA).

### Quantitative study

Following these screening procedures, a pre-specified 20% random sample of women (if women refused to participate, more were invited) from each site were invited to participate in exit surveys. Verbal consent was obtained from women who participated in the exit surveys, and they were assured that refusal to answer any question would not affect future follow-up or treatment at the clinic. All of the exit surveys were conducted face to face by women who were either health workers or nurses in the study clinic. Survey participants were asked closed-ended questions about their opinions, attitudes, health-seeking behaviors, potential barriers related to cervical cancer screening and self-collection procedures, and about their experiences participating in the study. Respondents were also asked if they collected a vaginal sample by themselves. If they answered “yes,” they were asked additional questions about their concerns (if any) of providing such a sample, asked to rate how easy or difficult it had been to collect the sample on a five-point Likert scale from “very difficult” to “very easy” (they were further categorized into “easy,” “neutral,” and “difficult”) and asked if they had chosen to self-collect a vaginal sample or asked the provider to do so. Finally, all respondents were asked about their opinions regarding additional aids needed to facilitate self-collection of vaginal samples and whether they would consider participating in future cervical cancer testing/screening opportunities. All analyses were conducted using Stata 12.0 (Statacorp, College Station, TX). Chi-square and Fisher’s exact tests were used to test differences in proportions between categories and study sites.

### Qualitative study

Preliminary analyses of the closed-ended exit surveys revealed a need to gain greater understanding of the acceptability and feasibility of women’s experiences with self-sampling. As a result, a highly focused follow-up study using qualitative research methods was designed. Studies were conducted in India, Nicaragua, and Uganda. In all three sites, semi-structured qualitative interviews (SSIs) were conducted with the demonstration project health providers and participants. In addition, in Nicaragua and Uganda, the research teams organized focus group discussions (FGDs). All health providers involved in the demonstration study participated in the SSIs; eligible women returning to the clinic for further cervical cancer assessment and/or treatment were opportunistically selected to participate in SSIs and FGDs. Verbal consent was obtained from all health providers and women that participated in the SSIs and FGDs.

In all three sites, experienced qualitative research staff conducted all of the SSIs and FGDs. During the follow-up individual SSIs and FGDs, providers and women were asked to describe reasons why women do not access screening services, to describe in detail their personal experiences with vaginal self-sampling, and whether they preferred to collect a vaginal self-sample at home or in the clinic and why or why not. In-depth notes were taken during the SSIs and FGDs. Both the SSIs and the FGDs were audio recorded so that the note-taker and interviewer could refer to the recordings if they felt that anything was missing from the notes or if they had questions about the notes during analysis. All SSIs and FGDs transcriptions were translated to English and research teams in each country provided in-depth session reports, and an overall summary report of their impressions and insights after having completed all of the SSIs and FGDs.

The in-depth session notes for SSIs and FGDs from each site were independently analyzed by a qualitative research specialist at PATH headquarters in the United States. Common themes and/or issues were grouped together and frequencies were noted in order to highlight key issues. These were shared and validated with local research teams. As this was a qualitative study, the frequencies were not reported. However, direct quotations were pulled from the session reports when they were illustrative of the issues.

## Results

Among the total 19,340 eligible and completely screened women from India, Nicaragua, and Uganda, 3,863 completed the exit survey, yielding an overall response rate of 20.0%. The response rates varied by site: rural Uttar Pradesh, India (18.7%); urban Hyderabad, India (22.3%); Nicaragua (15.5%); and Uganda (19.3%). A total of 20 providers and 82 women enrolled in the study participated in the SSIs and FGDs (Table 1). Among these, 37 were from Hyderabad, 33 from Nicaragua, and 32 from Uganda. Due to funding constraints, no components of the qualitative study were conducted in Uttar Pradesh.

**Table 1:** Qualitative study participantsSSIs: semi-structured interviews; FGDs: focus group discussions

	**Participant SSIs**		**Provider SSIs**	**Participant FGDs**		**Total respondents**
	**Self-sampling ACCEPTORS**	**Self-sampling REFUSERS**		**# of sessions**	**Total # of participants**	
**Hyderabad**	25	7	5	0	0	37
**Nicaragua**	5	5	10	2	13	33
**Uganda**	10	0*	5	2	17	32

*Very few START-UP study participants in Uganda refused self-sampling and no refusers returned to the clinic for follow-up and/or treatment during the qualitative research period.

### Women’s experience with self-sampling

**Figure 1:**
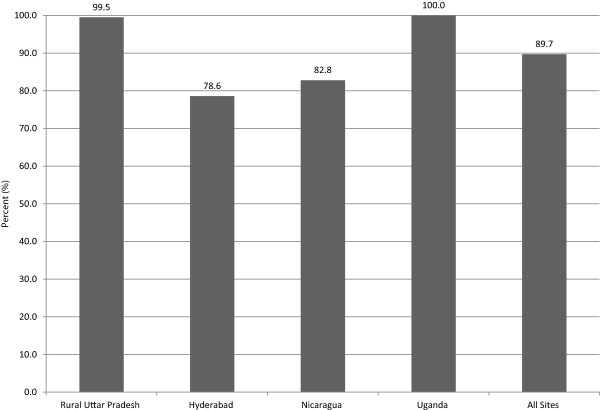
Percent of women who provided a self vaginal sample, by study site and all sites (N = 3464).

In all four sites, nearly 90% of surveyed women reported providing a self-collected vaginal sample (Figure 1). This was the highest in Uganda (100%) and rural Uttar Pradesh (99.5%), as compared to Nicaragua (82.8%) and Hyderabad (78.6%).The following quotations from interviewed providers in Uganda validate the observed high rate there:

“Generally speaking, most of the women were more than willing to do self-sampling. They were glad to do it and to become involved in their own screening.” —Provider, Uganda

“To me I think self-sampling is easily accepted as long as women are provided with sufficient information and they are given assurance about the safety of the brush.” —Provider, Uganda

A project nurse explained the process undertaken to prepare women for self-sampling in Uganda this way: Study participants first attended a short talk, augmented with a flipchart and/or video to illustrate the self-sampling process. They were shown a sample *care*HPV™ collection brush and asked to feel how soft it was. Later, women were taken to a private room where they were given instructions on how to use the brush to obtain a sample:

“I told them to insert the brush deep until it met resistance, then I would tell them to rotate the brush 3 to 5 times.” —Provider, Uganda

Usually the provider would remain in the room, facing away, in case they needed any additional help while gathering the sample.

### Ease and preference of self-sampling

More than half of surveyed respondents in each site reported that it was relatively easy to take a self-collected vaginal sample (*p* < 0.001) (Table 2). Women in rural Uttar Pradesh (93.1%), Hyderabad (95.5%), and Uganda (64.5%) preferred self-collected vaginal sampling, as compared to provider-collected cervical-sampling (*p* < 0.001). On the other hand, women in Nicaragua did not appear to have a preference: 50% preferred self-collected vaginal sampling, and the other half preferred provider-collected cervical-sampling. Regardless, women were positive about a screening test that did not involve a speculum exam and, for the most part, were satisfied with the experience, especially of having taken the sample themselves:

**Table 2:** Attitudes (concerns, ease, and preference) regarding self-sampling, among women who provided a self-collected vaginal sample (number and percent)

	**All sites (N = 3,464)**	**Rural Uttar Pradesh (N = 937)**	**Hyderabad (N = 912)**	**Nicaragua (N = 704)**	**Uganda (N = 911)**	***P *** **value**^**‡**^
Concerns about taking self vaginal sample*^†^						
Hurting oneself	1,804 (55.1)	374 (40.0)	793 (87.8)	355 (65.1)	282 (31.5)	<0.001
Not getting a good sample	822 (25.1)	154 (16.5)	79 (8.7)	165 (30.3)	424 (47.4)	<0.001
Dropping the brush/equipment to collect the sample	138 (4.2)	24 (2.6)	49 (5.4)	3 (0.6)	62 (6.9)	<0.001
Other	26 (0.8)	3 (0.3)	0 (0)	20 (3.7)	3 (0.3)	<0.001
No concern	561 (17.1)	383 (41.0)	0 (0)	22 (4.0)	156 (17.4)	<0.001
**Ease of taking self vaginal sample**						
Easy	2,597 (75.0)	672 (71.7)	489 (53.6)	619 (88.3)	817 (89.7)	<0.001
Neutral	704 (20.3)	260 (27.8)	300 (32.9)	53 (7.6)	91 (10.0)	
Difficult	160 (4.6)	5 (0.5)	123 (13.5)	29 (4.1)	3 (0.3)	
No response	3 (0.1)	0 (0)	0 (0)	3 (0.4)	0 (0)	
**Preferred collector of vaginal sample**						
Self	2,683 (77.5)	872 (93.1)	871 (95.5)	352 (50.0)	588 (64.5)	<0.001
Provider	781 (22.5)	65 (6.9)	41 (4.5)	352 (50.0)	323 (35.5)	

*Categories are not mutually exclusive; Women could select more than one response category.

^†^Percent’s are based on how many women answered the question; women that did not answer the question were excluded (All sites (N = 150); Rural Uttar Pradesh (N = 1); Hyderabad (N = 0); Nicaragua (N = 149) and Uganda (N = 0)), and women that checked more than one response were accounted for (All sites (N = 37); Rural Uttar Pradesh (N = 2); Hyderabad (N = 9); Nicaragua (N = 10) and Uganda (N = 16)), resulting in a sample size of: All sites (N = 3,277); Rural Uttar Pradesh (N = 934); Hyderabad (N = 903); Nicaragua (N = 545) and Uganda (N = 895)).

^‡^*P* value comparing the distribution among study sites.

“I did not have any difficulties inserting the brush. It is painless, better than having a medical person tell you to lie down on a bed, open your legs wide for visual inspection.” —Study participant, Uganda

“The women who have done this test should tell about their experiences to other women so that they may come forward and do this test.” —Study participant, India

### Concern’s about self-sampling

Many surveyed women initially reported having concerns with self-sampling (Table 2). Being worried about hurting oneself was the principal concern among women in Hyderabad (87.8%) and Nicaragua (65.1%), whereas women in Uganda reported being most concerned about not getting a good sample (47.4%) (*p* < 0.001); these were the two most common worries in all sites. The following quotations illustrate their experiences:

“I was worried that it might be painful, but there was no pain at all.” —Study participant, India

“The women asked “did I do it well?” So I showed them the secretions on the brush, and praised them for doing a good job.” —Provider, Nicaragua

Additional concerns included unwillingness to touch the genital region and/or the inability of women to see their own genital area while doing the procedure.

“Rural women are more shy about touching themselves than urban women, but will be more accepting of self-sampling than urban women.” —Provider, Nicaragua

“Most women accepted self-sampling, but for those who did not, the main reason they gave was not wanting to touch themselves.” —Provider, Uganda

“To take the sample from that particular area is not visible to me. I may not do it correctly, and may hurt myself. I tried doing but could not do it.” —Study participant, India

In India in particular, several respondents repeatedly raised the issue of whether obese women would be able to reach far enough to collect the vaginal sample effectively.

Also, doubts with using the sample brush itself were raised, as illustrated below:

“Some women were concerned about inserting the brush too far into their vaginas and hurting themselves.” —Provider, Nicaragua

“When they hear the word “brush,” women think of a brush with stiff bristles like a toothbrush or scrub brush. So I show them the sample brush. I let them stroke their skin with it to feel how soft it is.” —Provider, Uganda

Given these concerns, some interviewed respondents expressed that they would prefer a provider to collect the samples:

“I do not know where the small stick can go.” —Study participant, Nicaragua

“I prefer the medical person to do the sampling because she will see where she is inserting.” —Study participant, Uganda

“Everything was fine, but don’t want to do it by myself.” —Study participant, India

### Preference of self sampling at a clinic

Some interview respondents expressed feeling more comfortable collecting the self-sample in a clinic than at home, not only because providers could collect the sample and offer treatment, if needed, but also because the clinics were clean and sanitary and provided privacy. Also, to some, sampling at home seemed inconvenient:

“Doing it from home? I don’t know, I worry that it will come back contaminated. I would prefer to have it done in hospital because it takes such a short time. It does not make much sense to take the kit home, take the sample and then bring it back to hospital.” —Study participant, Uganda

“May be disturbances when too many people are at home. There is no time and privacy.” —Study participant, India

“It would be best to let the woman do the self-sample in the clinic the first time, then after she has experience, she can do it at home in the future.” —Study participant, Nicaragua

Furthermore, a number of providers shared these opinions:

“The only problem that I see is the ability of these women to take the specimen correctly even when the information has been provided. Therefore, I would not recommend for women to do this at home. I will recommend that the specimen is collected at a health facility. If there is need for privacy, you provide a room for the woman to do the self-sampling. I do not think they need to take the kit home—there is no need for that.” —Study provider, Uganda

### Reasons for not self-sampling

**Figure 2:**
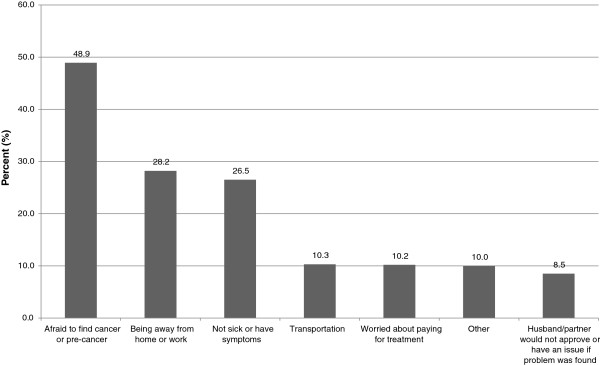
Women’s concerns for participating in the overall demonstration study (N = 3863).

We also interviewed women who chose not to provide a self-sample. Among survey participants, 399 (10.3%) of women across all sites reported that they did not provide a self-collected vaginal sample. When asked why they had made that choice, 44.9% of these women were worried about hurting themselves, 23.1% expressed fear of dropping the brush/equipment to collect the sample, and 8.0% were concerned about not being able to take a good sample (data not shown).Survey participants were also asked whether they had had concerns about participating in the overall demonstration study. Results show that the top three primary concerns were: being afraid to find cancer or pre-cancer (48.9%), being away from home or work (28.2%), and not currently feeling sick or not having any symptoms (26.5%) (Figure 2). When interviewed respondents were asked why other women may not access cervical cancer screening and treatment services, they reported: fear of positive screening results, fear of needing treatment, or fear of “losing part of the womb” to a biopsy or surgery; lack of information about screening; fear of pain during screening or distaste for pelvic exams due to the discomfort associated with the use of a speculum; having to wait a long time for services or for results (especially for Pap smears) and the need for repeat visits; refusal by husband or family elders to give permission to go to the clinic or be examined by a male provider; and shyness or embarrassment about being examined by a male provider.

### Aids to facilitate self-sampling

When asked what could aid women to provide a self-sample, 52.6% of all the women surveyed reported that getting assistance with the procedure from staff would be the primary aid needed, followed by more pictures (30.6%), and use of a doll or model (25.9%) (Table 3).

**Table 3:** Women’s opinions on future aids needed to facilitate self-collection of vaginal sample (number, percent)

	All sites (N* = 3,195)	Rural Uttar Pradesh (N* = 917)	Hyderabad (N* = 863)	Nicaragua (N* = 705)	Uganda (N* = 710)	***P *** **value**^**‡**^
**Aids needed to ease self-collection in the future†**						
Staff help	1,679 (52.6)	479 (52.2)	496 (57.5)	311 (44.1)	393 (55.4)	<0.001
More pictures	977 (30.6)	277 (30.2)	415 (48.1)	119 (16.9)	166 (23.4)	<0.001
Doll/model	828 (25.9)	130 (14.2)	399 (46.2)	35 (5.0)	264 (37.2)	<0.001
None	710 (22.2)	39 (4.3)	154 (17.8)	252 (35.7)	265 (37.3)	<0.001

*Sample size’s are based on how many women answered the question; Of the total number of women ((All sites (N = 3,863); Rural Uttar Pradesh (N = 942); Hyderabad (N = 1,160); Nicaragua (N = 850) and Uganda (N = 911)), women that did not answer the question were excluded (All sites (N = 181); Rural Uttar Pradesh (N = 21); Hyderabad (N = 0); Nicaragua (N = 139) and Uganda (N = 21)), and women that checked more than one response were accounted for (All sites (N = 487); Rural Uttar Pradesh (N = 4); Hyderabad (N = 297); Nicaragua (N = 6) and Uganda (N = 162)).

^†^Categories are not mutually exclusive; Women could select more than one response category.

^‡^*P* value comparing the distribution among study sites.

## Discussion

Among surveyed women in India, Nicaragua, and Uganda, of the 90% that provided a self-collected sample for cervical cancer screening, a vast majority preferred vaginal sampling, whether done by themselves or a provider. Given that these findings are consistent with previous reviews, [11,12] self-sampling is a viable option for cervical screening, especially in low-resource settings, and therefore can be incorporated into early detection programs to increase coverage. These findings are particularly important given that screening women with an HPV-DNA test once at 35 years of age has the potential to reduce the lifetime risk of cervical cancer by 36% [2]. This is especially encouraging for clinics in developing countries with limited resources, as they can prioritize their efforts to efficiently screen women and focus on treating those that test positive.

The high rate of vaginal self-sampling that we observed in this study, particularly in Uganda, illustrates that providers have considerable influence on self-sampling. Ugandan providers prepared women for self-sampling through health education, allowing women to touch and feel a sample brush, and were present to provide support during the self-collection process. Once they realized that some women imagined stiff hair brushes or toothbrushes when they heard the word “brush” (and worried that such a brush would hurt them), the staff began to emphasize that it was a “soft brush.”

Even though we found that most interviewed women were positive about vaginal self-sampling, many also expressed that they would prefer a provider to collect the vaginal sample rather than collecting it themselves. This caused us to wonder what it was that attracted women to the self-sampling option. Unfortunately we had not incorporated probing questions to determine whether women were most keen on the idea of taking the sample themselves (for privacy) or whether they simply preferred to not undergo a speculum exam and were happiest to have someone else take the vaginal sample for them. Given that the acceptability of pelvic examinations among women is low (51%) [15], that quite a few respondents in the follow-up interviews mentioned concerns about the quality of taking a sample themselves, and that many women shared other, practical reasons why self-sampling—especially away from the clinic—was problematic, it is tempting to speculate that vaginal sampling was the draw. Further research into that question would be useful. If that speculation is borne out, we can visualize scenarios in which trained, female village health workers or volunteers could be mobilized for mass sample collection. For example, a team of five or six experienced “vaginal samplers,” given private spaces for each of them to work in such as temporarily adapted school rooms, could collect hundreds of samples in just a few hours without the need for speculums or examination tables. This could be a major factor in finally achieving screening at scale in low-resource settings.

Our findings show that women were not inclined toward vaginal self-sampling at home. Research in other settings indicates mixed results. One study in China found that a majority of women preferred to do self-sampling at the clinic rather than at home [21]. A study among Ugandan women from a low-resource district in Kampala found that participation in a program involving self-collection was positively associated with health workers dropping off swabs at their homes [18]. In South India, women had higher rates of participation in self-sampling for cervical cancer screening at home (71.5%) than at the clinic (53.8%) [22]. In yet another study, women in Mexico preferred to do the test in a clinic (76.8%) [23]. When asked why, these women indicated that a provider could clarify their questions, they felt more comfortable if a doctor/nurse was present, and the provider could do the test if needed.

As in other studies, many women in our study reported challenges associated with vaginal self-sampling. Women in our study reported their main concerns were fear of pain and administering the test correctly; however, most of them did not report religious or cultural barriers as hindering participation, as previously reported [13,17-19,23]. Further barriers to screening were expressed by women in our study as fear of finding out that they had cancer, not feeling ill, and being away from home or work. Similar results were found in an acceptability study in Mexico, where 70% reported lack of symptoms and 61% of women reported lack of time as major barriers to cervical cancer screening [23]. These results highlight the continuing need for community education and awareness outreach programs that dispel fears/concerns and clarify misconceptions about screening and that mobilize the entire community to encourage women to participate in cervical cancer screening programs.

Our study had some limitations. First, aside from participant age, socio-demographic information was not collected. Hence, we were unable to assess the influence of education, economic status, and other factors on the acceptability of self-sampling or vaginal sampling. Second, as our study was part of a larger demonstration project, the women who participated in our study had undergone four cervical cancer screening procedures, and thus may not be representative of their respective communities and/or countries. In addition, as sampling for the survey, interviews and FGDs was opportunistic, these findings should not be assumed to be generalizable to larger populations. Furthermore, as participants for interviews and FGDs were selected from women returning to the clinic for further evaluation and/or treatment, their views and opinions may not be representative of all women and therefore should be interpreted with caution. These limitations notwithstanding, our findings do address women’s experiences and concerns with cervical cancer screening and provided valuable insights for vaginal self-sampling acceptability and strategies, in low-resource settings.

## Conclusion

In conclusion, despite the cultural diversity of women in India, Nicaragua and Uganda, majority provided a self- or provider-collected vaginal sample. In a future study or during field implementation, we would recommend developing additional, culturally appropriate, educational aids such as pictures, dolls, or models. Because the language used to introduce the sample collection device is key to women’s acceptance of self-sampling, it is important to be sensitive in regards to terminology used to describe the test (e.g., routinely saying “soft brush” vs. “brush”). Further, future strategies to promote vaginal-sampling for cervical cancer screening programs could mobilize trained providers to collect vaginal samples from women, or to support women during self-sampling, assuming that a private room can be provided in a clinic, health post, mobile clinic, or temporary service site (such as a school). Finally, in order to increase the uptake of cervical screening, and because providers influence the acceptability and success of vaginal self-sampling, it is imperative that services consistently offer self-sampling and that providers’ be comprehensive when presenting screening options.

## Competing interests

PB, SW, JLL, JLW, and PP have no competing interests. JJ was the director of the demonstration study and received the *care*HPV tests as a donation from the manufacturing company (QIAGEN).

## Authors’ contributions

JJ and JLL participated in the design of the study and managed the conduct of the study in all the sites. SW oversaw the design and data collection of the qualitative data, analyzed and compiled the qualitative results. PB analyzed and compiled the quantitative results and drafted the initial manuscript. All authors made substantial contributions to the interpretation of the results and assisted in drafting the manuscript. All authors read and approved the final version of the manuscript.

## Pre-publication history

The pre-publication history for this paper can be accessed here:

http://www.biomedcentral.com/1471-2458/14/596/prepub
